# A Novel Bidirectional Interaction between endothelin-3 and Retinoic Acid in Rat Enteric Nervous System Precursors

**DOI:** 10.1371/journal.pone.0074311

**Published:** 2013-09-09

**Authors:** Jonathan M. Gisser, Ariella R. Cohen, Han Yin, Cheryl E. Gariepy

**Affiliations:** 1 The Center for Molecular and Human Genetics, the Research Institute at Nationwide Children’s Hospital, Columbus, Ohio, United States of America; 2 Department of Pediatrics, the Ohio State University, Columbus, Ohio, United States of America; 3 The Biostatistics Shared Resources, Nationwide Children’s Hospital, Columbus, Ohio, United States of America; Laboratoire de Biologie du Développement de Villefranche-sur-Mer, France

## Abstract

**Background:**

Signaling through the endothelin receptor B (EDNRB) is critical for the development of the enteric nervous system (ENS) and mutations in endothelin system genes cause Hirschsprung’s aganglionosis in humans. Penetrance of the disease is modulated by other genetic factors. Mutations affecting retinoic acid (RA) signaling also produce aganglionosis in mice. Thus, we hypothesized that RA and endothelin signaling pathways may interact in controlling development of the ENS.

**Methods:**

Rat immunoselected ENS precursor cells were cultured with the EDNRB ligand endothelin-3, an EDNRB-selective antagonist (BQ-788), and/or RA for 3 or 14 days. mRNA levels of genes related to ENS development, RA- and EDNRB-signaling were measured at 3 days. Proliferating cells and cells expressing neuronal, glial, and myofibroblast markers were quantified.

**Results:**

Culture of isolated ENS precursors for 3 days with RA decreases expression of the endothelin-3 gene and that of its activation enzyme. These changes are associated with glial proliferation, a higher percentage of glia, and a lower percentage of neurons compared to cultures without RA. These changes are independent of EDNRB signaling. Conversely, EDNRB activation in these cultures decreases expression of RA receptors β and γ mRNA and affects the expression of the RA synthetic and degradative enzymes. These gene expression changes are associated with reduced glial proliferation and a lower percentage of glia in the culture. Over 14 days in the absence of EDNRB signaling, RA induces the formation of a heterocellular plexus replete with ganglia, glia and myofibroblasts.

**Conclusions:**

A complex endothelin-RA interaction exists that coordinately regulates the development of rat ENS precursors *in vitro*. These results suggest that environmental RA may modulate the expression of aganglionosis in individuals with endothelin mutations.

## Introduction

During embryonic development, vagal neural crest cells must migrate caudally, proliferate, differentiate and organize into ganglionated plexuses in order to form a fully functional enteric nervous system (ENS) [1,2]. Failure to do so in a spatiotemporally regulated manner results in varying lengths of terminal aganglionosis, which manifests as the common congenital disorder Hirschsprung’s disease. Mutations in genes encoding the endothelin (EDN) and RET (rearranged during transfection) signaling pathways account for the majority of cases of Hirschsprung’s disease [3,4]. Mutations in the genes encoding the EDN receptor B (EDNRB), its ligand EDN3, and the transcription factor SOX10 cause Waardenburg-Shah syndrome (also known as Waardenburg syndrome type IV), comprised of pigment cell abnormalities and Hirschsprung’s disease. The *EDNRB* gene encodes a G-protein coupled receptor that is expressed on ENS precursors during development [5,6]. EDN3, is a 22 kDa peptide that is activated by the EDN converting enzyme 1 (ECE1) [7,8]. It is expressed in a spatiotemporally controlled manner by the gut mesenchyme, with expression preceding the arrival of precursor cells and continuing during their migration through the hindgut [5,9]. *In vitro*, EDN3 inhibits neuronal differentiation and stimulates proliferation of enteric neuronal precursors [10,11]. Rodents carrying null mutations in *Ednrb* exhibit colonic aganglionosis attributable to an early modest reduction in the number of enteric neural crest-derived stem cells and migration failure in the hindgut [6,10–15]. The variable length of aganglionosis in rodents carrying *EDN3* or *Ednrb* mutations and the low penetrance of the Hirschsprung’s phenotype in humans carrying *EDN3* or *EDNRB* mutations is partially explained by studies showing a genetic interactions between *Ednrb*, *Ret*, and *Sox10* mutations [16,17]. Environmental factors that influence EDNRB signaling in ENS development have not been investigated.

Retinoic acid (RA) is a derivative of dietary vitamin A that is generated from retinaldehyde in its final synthetic step by three distinct retinaldehyde dehydrogenases (RALDH), all of which are expressed in the fetal bowel [18,19]. Retinaldehyde dehydrogenase 2 (RALDH2), is expressed in the gut mesenchyme during development, but its regulation is poorly understood [20,21]. RA forms a complex with its cognate RA receptors (RAR α, β, and γ), translocates to the nucleus, and binds to RA receptor elements encoded in the genome to affect gene transcription [22]. RA is inactivated by the cytochrome P450 26 family of enzymes [23,24]. *Cyp26a1* transcripts are detected in the outer mesenchyme of the murine esophagus and stomach during development [25]. *Cyp26a1* transcripts are also found in the mesenchyme and ENS of the developing small intestine, and CYP26A1 may be critical for maintaining RA gradients in the developing embryo [21,26]. RA affects multiple processes in ENS development: Excess RA induces a delay in ENS precursor migration into the intestine caudal to the cecum in intestinal explant cultures [27] – a phenomenon that mimics EDNRB signaling deficiency. However, a paucity of RA *in utero*, induced by maternal dietary deprivation of vitamin A in susceptible rodents, is also associated with aganglionosis [28]. A role for RA in migration is also supported by *in vitro* evidence that RA modulates neural crest cell polarity and cytoskeletal arrangement. In addition, RA enhances the proliferation of RET+ ENS neuronal precursors and enhances neuronal differentiation of this subset of cells *in vitro* [21].

EDNRB and RA signaling must be tightly regulated to produce normal ENS development [5,15,29], but their combined effects on the development of the ENS are unknown. Work in other systems suggests that RA reduces EDN signaling. RA suppresses *Edn 1* expression in cultured hepatic stellate cells, a prostate cancer cell line, renal glomerular cells and endothelial cells [30–33]. To date, however, a relationship between RA and *EDN3* or *Ednrb* gene expression has not been established. We studied the relationship of EDNRB and RA signaling on immunoselected rat p75-neurotrophin receptor expressing (p75^NTR^+) ENS precursors. These cells are neural crest cells that form the neurons and glia of the ENS and are commonly cultured *in vitro* as a model of ENS development [10,21,34–36]. We report that the combination of exogenous EDN3 and RA exerts unique effects on these ENS precursors compared to the addition of each compound alone. Specifically, we demonstrate that RA and EDN3 exert opposing effects on *in vitro* enteric glial development and *Sox10* expression. We further suggest that RA is a suppressor of EDNRB signaling, that EDN3 disrupts RA signaling and metabolism, and that this interaction results in dramatic morphologic differences in these *in vitro* cultures. 

## Materials and Methods

### Ethics Statement

This study conformed to the Guide for the Care and Use of Laboratory Animals published by the U.S. National Institutes of Health (NIH Publication No. 85–23, revised 1996) and was approved by the Nationwide Children’s Hospital Institutional Animal Care and Use Committee (IACUC protocol numbers AR09-00042 and AR11-00012, Institutional Animal Assurance Number A3544-01)

### ENS precursor cell isolation and enrichment

Rat ENS precursors were isolated from inbred Wistar-Kyoto (WKY-NHsd) rats using a method adapted from Sato and Heuckeroth [21]. Briefly, prenatal gut tissue from the stomach to the colon, was dissected from rat embryos on gestational day E14.5, and dissociated into a cell suspension by incubating with collagenase IV (10 mg/ml; Gibco) and 0.05% trypsin-EDTA (50%; Gibco), treating with DNase type I (250µg/ml), and passing the suspension through a nitex filter (38 micron pore) to remove debris. Cells were then labeled with an antibody to the p75^NTR^ (1:200, clone IgG 192, Calbiochem) followed by a secondary antibody conjugated to magnetic beads (Miltenyi Biotech). Cells bound by magnetic beads were then positively magnetically selected in a MACS separation column (Miltenyi Biotech), and eluted outside of the magnetic field. Selected cells were used for all subsequent experiments. The purity of the selected cell suspension was assayed by determining the proportion of p75^NTR^ expressing cells after elution. The cell suspension (5x10^5^ cells/ml) was cytocentrifuged, fixed in 4% formaldehyde and immunolabelled with antibody to p75^NTR^ (1:200, clone IgG 192, Calbiochem) followed by secondary fluorescent antibody-labeling with anti-mouse IgG Alexa Fluor 488. Selected cells consistently comprised around 10% of the total cell yield (data not shown), and 77% of these cells retained p75^NTR^ positivity when re-labeled within two hours of isolation. In contrast, only 7% of the non-selected of cells were p75^NTR^+. These data contrast with previous work performed in mice and rats where the proportion of p75^NTR^+ cells among selected cells approached 90% [10,21], possibly reflecting interspecies and/or subtle methodologic differences. As such, we assume that our cell population, while not homogenous, still contains a large enriched fraction of ENS precursors.

### Medium and culture conditions

For all experiments, we modified the medium used by Kruger et al. by omitting chick embryo extract and vitamin A [15]. The medium consists of DMEM-low glucose medium (Gibco) supplemented with Penicillin/streptomycin (1X, BioWhittaker), Normocin (50µg/ml, InvivoGen), N2 (1%, Gibco), B27 without vitamin A (2%, Invitrogen), β-mercaptoethanol (50µM, Sigma-Aldrich), Glial Cell Line-Derived Neurotrophic Factor (50ng/ml, R&D systems), and basic Fibroblast Growth Factor (20ng/ml, R&D systems). Selected cultures were also treated with RA (117nM, Sigma-Aldrich), EDN3 (100nM, Calbiochem), and/or the selective antagonist of the EDNRB, BQ-788 (5µM, Sigma-Aldrich) in the following combinations: EDN3-RA-, EDN3+RA-, EDN3-RA+, and EDN3+RA+. The concentrations of RA and EDN3 used were adopted from Kruger et al., as a basis for comparison to their work [15]. In all cultures without EDN3, the EDNRB signaling inhibitor BQ-788 was added to inhibit endogenous EDN3 signaling. An analogous RA signaling inhibitor was not added in RA-free cultures because RA abundance in dissociated cell cultures in the absence of added vitamin A or RA has previously been shown to be negligible [21]. Once added, all compounds were present for the life of the cultures, within the limits of their metabolism, as the medium was not changed for the duration of the culture. All culture dishes were pre-coated with poly-D-lysine (150µg/ml, Biomedical Technology) and laminin (20µg/ml, Invitrogen), and cells were cultured in a reduced oxygen environment in a chamber (Billups-Rothenberg) equilibrated with a gas mixture containing 1% O_2_/6% CO_2_/balance N_2_, generating an actual concentration inside the chamber of 3–6% oxygen [37].

### RNA isolation and gene expression profiling

ENS precursors were cultured and supplemented with different combinations of EDN3 and RA as above, in 6-well culture plates (Corning Costar) for 3 or 14 days. After culture, adherent cells were washed with PBS, and RNA was isolated using the RNeasy RNA extraction kit (Qiagen) per the manufacturer’s instructions. Genomic DNA was eliminated during the column purification using RNase-free DNase (Qiagen). cDNA was generated from total RNA using the Maxima First Strand cDNA Synthesis Kit (Fermentas) and quantitative PCR was performed using Maxima Probe/ROX qPCR master mix (Fermentas) with the following conditions: 95°C for 10 min x1; 95°C for 15 sec, 60°C for 1 min for 40 cycles. Intron-spanning primers pairs with a Tm of 59-60^o^C generating amplicons of 63-122 base pairs were synthesized by Integrated DNA Technologies Incorporated (Table 1). Amplicon-specific fluorescent probes were purchased from Roche. Using the ΔΔCT method, raw CT values were normalized to β-actin. EDN3-RA- cultures were assigned a value of 1, and all expression data are reported relative to these values. Data are averages of 3-4 biological replicates and 2 technical replicates. –RT controls were also performed.

**Table 1:** Primer – probe pairs used to amplify RA and EDN related genes.

gene	forward	reverse	Probe #
***Rara***	5'-ggcatgtccaaggagtcg-3'	5'-cgcaccttctcgatgagttc-3'	10
***Rarb***	5'-agcccaccaggaaacctt-3'	5'-gcactggaattcgtggtgta-3'	53
***Rarg***	5'-ctcatcaccaaggtcagcaa-3'	5'-atctgcgctggagttcgt-3'	53
***Raldh2***	5'-ggacgcttctgaaagaggac-3'	5'-ccgccatttagtgattccat-3'	120
***Cyp26a1***	5'-gagagaggagagaggctggata-3'	5'-ggctgcactggctgtagttt-3'	49
***Ednrb***	5'-ctgttggcttccccttcac-3'	5'-tgtagtccaaaaccagcaaaaa-3'	18
***Edn3***	5'-agaagcaggagactggaggtc-3'	5'-cagctaaggctggtggactt-3'	41
***Ece1***	5'-cggaggacagcaagaacatag-3'	5'-caggctctcctcgaatgc-3'	80
***Ret***	5'-cacagccttccgtctgaaa-3'	5'-tctgggaggcgttttcttt-3'	76
***Sox10***	5'-atgtcagatgggaacccaga-3'	5'-gtctttggggtggttggag-3'	21

### Immunocytochemistry

Cells were cultured in 8-well chamber slides (Thermo Scientific) at low density (1000 cells/well) with different combinations of EDN3 and RA as above. After three or fourteen days, cells were fixed in 3.7% paraformaldehyde, permeabilized in triton x-100, and incubated with primary antibody overnight at 4^o^C. After washing, secondary antibody was added. Primary antibodies: rabbit anti-peripherin (1:1000, Chemicon), mouse anti-smooth muscle actin (SMA) (1:400, Sigma), mouse anti-S100β (1:500, Sigma). Secondary antibodies (Invitrogen): anti-rabbit Alexa Fluor 546, and anti-mouse Alexa Fluor 488, anti-rabbit Alexa Fluor 488. Nuclei were counterstained with prolong gold antifade with DAPI mounting medium (Invitrogen).

### Proliferation assay

The click-it EDU proliferation assay (Invitrogen) was performed proliferation assay was performed according to the manufacturer’s instructions. Cells were cultured for 66 hours, followed by EDU (10µM) incorporation for 6 hours. After incorporation, cells were washed, fixed and labeled with Alexa Fluor 488 or 594 click-it reagent. Immunolabelling for different cell lineages and DAPI counterstaining were then performed as described in the immunocytochemistry section.

### Microscopy

Cells were visualized with a Zeiss AxioScope A1 epifluorescent microscope with a Zeiss Axiocam HRc digital camera and images were recorded using Axiovision software. An AMG EVOS “FL” Epifluorescence microscope was used for some experiments. All images were processed and analyzed using NIH ImageJ software, and ImageJ was used for manual cell counting.

### Statistical analysis

All quantitative experiments were repeated 3-9 times. To evaluate the individual contributions of exogenous EDN3 and RA, as well as any interaction effects between the two compounds, a two-way ANOVA (SigmaPlot) was used. The All Pairwise Multiple Comparison Procedure (Tukey Test) was used to determine the individual contributions of RA (comparing RA- vs. RA+) and EDN3 (comparing EDN3- vs. EDN3+) to changes observed in culture. Note that each comparison can be made in the presence or absence of the other compound. For instance, comparing EDN3-RA- to EDN3+RA- provides information about an EDN3 effect, as does comparing EDN3-RA+ to EDN3+RA+, but the latter is also in the presence of RA. The definition of an interaction is thus: The direction and/or magnitude of an observed change in response to one compound are affected by the presence of the other compound. A p value <0.05 is deemed significant in all experiments. Graphs were generated using Graphpad Prism 6. 

## Results

### RA and EDN3 have opposing effects on S100β+ cell prevalence

**Figure 1:**
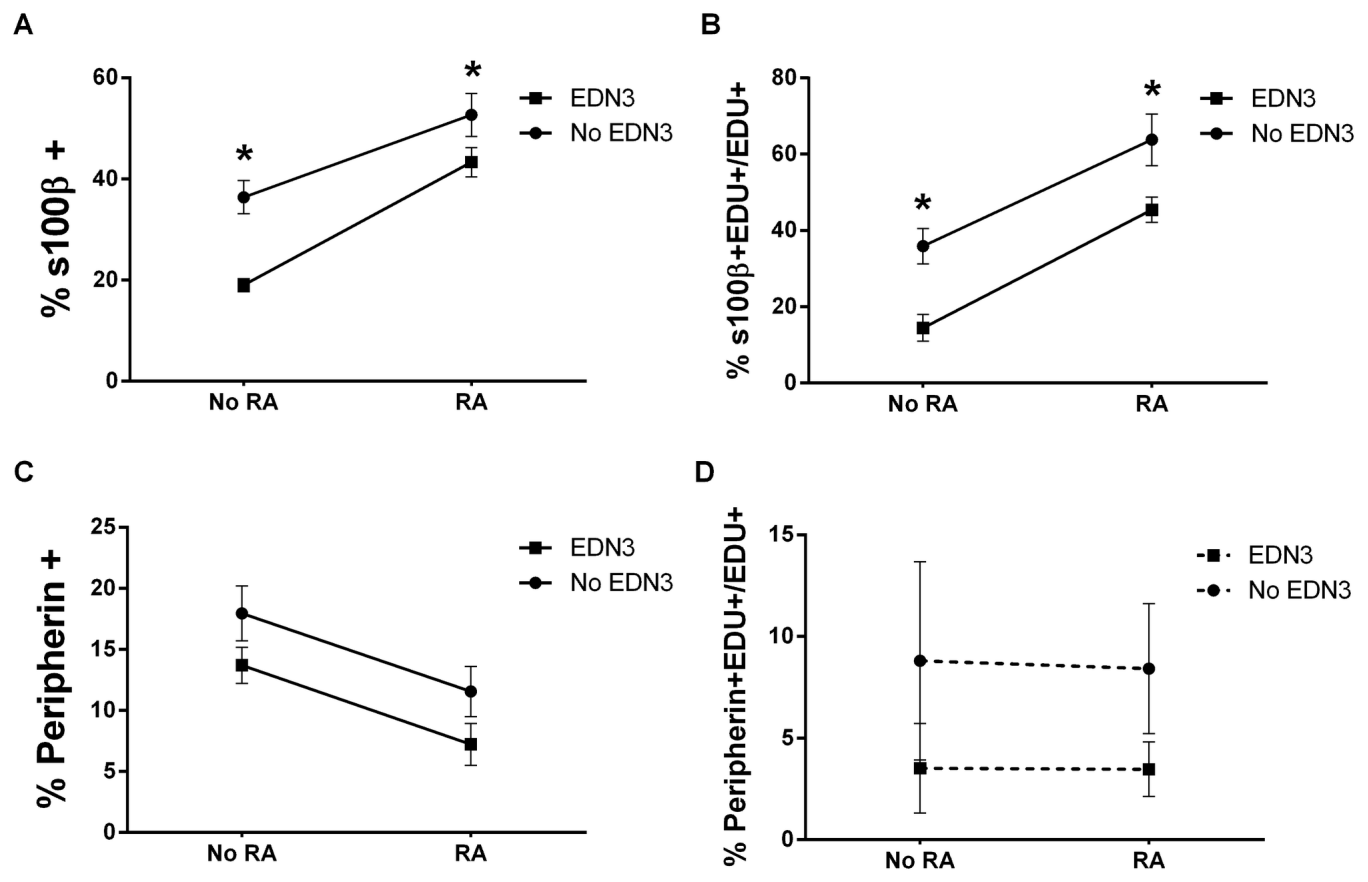
RA increases S100β+ and decreases peripherin+ cell prevalence while EDN3 decreases S100β+ cell prevalence. p75^NTR^+ cells were grown for 72 hours in the presence of RA, EDN3, RA with EDN3, or neither compound. When EDN3 was absent, the EDN signaling inhibitor BQ-788 was added to inhibit endogenous EDNRB signaling. The proportion (A and C) and proliferating fraction (B and D) of peripherin- and S100β-immunoreactive cells were quantified. Solid lines denote a statistically significant difference (p<0.05) between “No RA” and “RA”, and broken lines indicate a p value >0.05. Asterisk denotes a statistically significant difference (p<0.05) between “No EDN3” and “EDN3”. (A) The proportion of S100β+ cells was enriched by RA and decreased in response to EDN3 (B) RA increased, and EDN3 decreased the fraction of proliferating S100β+ cells. (C) the proportion of peripherin+ cells decreased in response to RA treatment, but EDN3 did not have an effect. (D) Neither RA nor EDN3 had an effect on proliferation of peripherin+ cells.

To determine how RA and EDN3 together and separately influence the *in vitro* cellular composition of the ENS, p75^NTR^+ immunoselected cells were isolated and exposed in parallel to four separate culture environments: 1) EDN3-RA- was devoid of exogenous RA, vitamin A, and EDN3 and the EDN signaling inhibitor BQ-788 was added to further inhibit any endogenous EDNRB signaling; 2) EDN3+RA-, where only EDN3 was added, permits EDNRB activation only; 3) EDN3-RA+, where RA was added with BQ-788, permitting RA activation only; and 4) EDN3+RA+, where EDN3 and RA were added and duel activation of EDNRB and RA is presumed to occur. These combinations permitted us to compare the effects of RA-signaling in the presence and absence of EDN3 and, conversely, the effects of EDN3 signaling in the presence and absence of RA. After 3 days of culture, the proportions of peripherin, SMA, and S100β immunoreactive cells were determined immunocytochemically (Figure 1 and Table 2). We found that the proportion of S100β+ cells, bearing a morphological resemblance to glia, was greatly enriched by RA. Comparing EDN3-RA- to EDN3-RA+, the proportion of S100β+ cells increased from 36.4% to 52.6% of the total cells in culture, a 45% increase (p<0.001). Similarly, the proportion more than doubled from 19.0 to 43.3% (p<0.001) when comparing EDN3+RA- to EDN3+RA+. Thus, regardless of whether EDN3 is present, RA is associated with a statistically significant increased percentage of S100β+ cells. In contrast, a comparison of EDN3-RA- to EDN3+RA- reveals a net decrease in S100β+ cell prevalence from 36.4% to 19.0%, a 47% decrease (p<0.001). Likewise, comparing EDN3-RA+ to EDN3+RA+ reveals a net decrease in S100β+ cell prevalence from 52.6% to 43.3%, an 18% decrease (p=0.043). Collectively, these data demonstrate that RA and EDN3 treatment have opposite effects on the composition of 3-day ENS precursor cultures, with RA enriching S100β+ cells and EDN3 depleting them.

**Table 2:** Changes in cell composition in response to RA and EDN3.

	Comparison of mean proportion of immunolabelled cells (p value)		
	%peripherin+	%SMA+	%S100β+
**RA- vs. RA+ (EDN3-)**	**17.9 vs. 11.5 (p=0.023)**	32.4 vs. 23.4	**36.4 vs. 52.7 (p=0.001)**
**RA- vs. RA+ (EDN3+)**	**13.7 vs. 7.2 (p=0.022)**	27.2 vs. 25.3	**19.0 vs. 43.3 (p<0.001)**
**EDN3- vs. EDN3+ (RA-)**	17.9 vs. 13.7	32.4 vs. 27.2	**36.4 vs. 19.0 (p<0.001)**
**EDN3- vs. EDN3+ (RA+)**	11.5 vs. 7.2	23.4 vs. 25.3	**52.7 vs. 43.3 (p<0.043)**
**Interaction present?**	No	No	No

This table summarizes the effects of RA and EDN3 on the cell composition of ENS precursor cultures after 3 days. Immunoselected cells were cultured in the presence of RA, EDN3, RA with EDN3, or neither compound. When EDN3 was absent, the EDN signaling inhibitor BQ-788 was added to inhibit endogenous EDNRB signaling. The proportion of peripherin, SMA, and S100β immunoreactive cells as a percentage of all cells counted in each condition were compared. A two way ANOVA was employed to identify the contribution of each compound to changes in the proportion of immunolabelled cells. Significant changes are in bold and p values are noted. Data is the summary of nine biological replicates, and ten random microscope fields were counted and summed per condition. Abbreviations: SMA = α-smooth muscle actin; EDN3 = endothelin-3; RA = Retinoic acid;

### RA decreases the prevalence of peripherin+ cells

Concomitant with the RA-associated increase in S100β+ cells in culture, there was a decrease in the proportion of peripherin+ cells, signifying differentiating neurons (Figure 1 and Table 2). With the addition of RA, the proportion of peripherin+ cells decreased from 18 to 11.5% (EDN3-RA- *vs.* EDN3-RA+), a 35% decrease (p=0.023), and from 13.7 to 7.2% (EDN3+RA- *vs*. EDN3+RA+), a 47% decrease (p=0.022). EDN3 did not have a statistically significant effect on the percentage of peripherin+ cells and no combinations of EDN3 and RA affected the prevalence of SMA+ cells.

### RA increases, and EDN3 decreases, the proliferation of S100β+ cells

To understand the mechanism underlying the compositional differences in response to EDN3 and RA exposure, we performed an EDU incorporation assay to study proliferative differences (Figure 1 and Table 3). In the presence of RA, there was an increase in the fraction of proliferating (% EDU+/DAPI+) cells overall, without respect to lineage. EDN3 also promoted proliferation, but its effect was not significant in the presence of RA. Upon examination of specific lineages, RA promoted the proliferation of S100β+ cells: the proportion of EDU+S100β+ out of all EDU+ cells nearly doubled from 35.9 to 63.8% in response to RA (p<0.001) and tripled when EDN3 was also present (p<0.001). Conversely, the proportion of proliferating S100β+ cells decreased in response to EDN3 from 35.9 to 14.4% (p=0.006) in the absence of RA and from 63.8 to 45.4% (p=0.015) in the presence of RA. There were no significant changes in the proportion of proliferating peripherin+ and SMA+ cells in any culture condition. Collectively, these data demonstrate that RA stimulates the proliferation of S100β+ cells *in vitro*, and that EDN3 has an opposing effect on S100β+ proliferation thereby reducing the number of S100β+ cells in the culture.

**Table 3:** Lineage-specific and overall changes in proliferation in response to RA and EDN3.

	Comparison of mean proportion of proliferating cells (p value)			
	%EDU+/DAPI+	%EDU+peripherin+/EDU+	%EDU+SMA+/EDU+	%EDU+S100β+/EDU+
**RA- vs. RA+ (EDN3-)**	**25.8 vs. 45.1 (p<0.001)**	8.8 vs. 8.4	28.5 vs. 18.3	**35.9 vs. 63.8 (p<0.001)**
**RA- vs. RA+ (EDN3+)**	**33.0 vs. 48.8 (p<0.001)**	3.5 vs. 3.5	19.2 vs. 8.4	**14.4 vs. 45.4 (p<0.001)**
**EDN3- vs. EDN3+ (RA-)**	**25.8 vs. 33.0 (p=0.014)**	8.8 vs. 3.5	28.5 vs. 19.2	**35.9 vs. 14.4 (p=0.006)**
**EDN3- vs. EDN3+ (RA+)**	45.1 vs. 48.8	8.4 vs. 3.5	18.3 vs. 8.4	**63.8 vs. 45.4 (p=0.015)**
**Interaction present?**	No	No	No	No

This table summarizes the effects of RA and EDN3 on the proliferation of ENS precursor cultures after 3 days. Immunoselected cells were cultured in the presence of RA, EDN3, RA with EDN3, or neither compound. When EDN3 was absent, the EDN signaling inhibitor BQ-788 was added to inhibit endogenous EDNRB signaling. The overall proportion of proliferating cells (EDU+/DAPI+), and the proportion of proliferating cells of each lineage as a percentage of all proliferating cells (EDU+) were quantified and compared between the groups. A two way ANOVA was employed to identify the contribution of each compound to changes in the proportion of proliferating cells. Significant changes (p<0.05) are in bold. Abbreviations: SMA =α-smooth muscle actin; DAPI = 4',6-diamidino-2-phenylindole; EDU = 5-ethynyl-2´-deoxyuridine; EDN3 = endothelin-3; RA = Retinoic acid;

### RA and EDN3 interact to affect Sox10 expression

**Figure 2:**
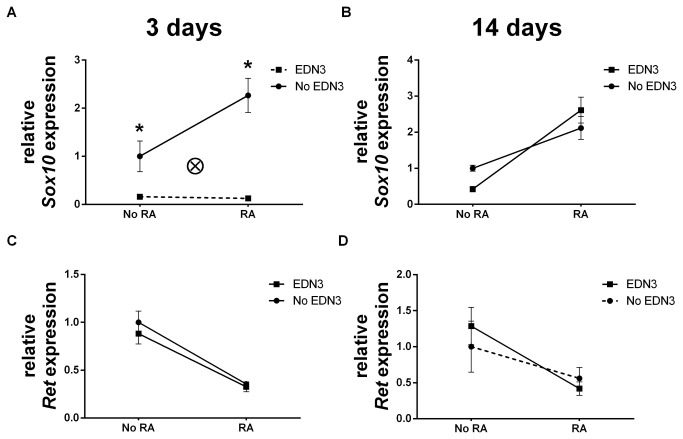
Culture in the presence of RA increases *Sox10*, but EDN3 suppresses this effect. p75^NTR^+ cells were grown for 72 hours in the presence of RA, EDN3, RA with EDN3, or neither compound. When EDN3 was absent, the EDN signaling inhibitor BQ-788 was added to inhibit endogenous EDNRB signaling. Relative *Sox10* (A–B) and *Ret* (C–D) mRNA levels were measured by quantitative RT-PCR after 3 (A, C) and 14 (B, D) days. Expression is normalized to β-actin and the -RA/-EDN3 condition was standardized to a value of 1. Solid lines denote a statistically significant difference (p<0.05) between “No RA” and “RA”, and broken lines indicate a p value >0.05. Asterisk denotes a statistically significant difference (p<0.05) between “No EDN3” and “EDN3”. (A) In the absence of EDN3, RA increased *Sox10* levels; however, the concurrent addition of EDN3 abolished this phenomenon. There was a statistically significant interaction (ⓧ; p=0.019) between EDN3 and RA in their effect on *Sox10* levels at 3 days. This interaction is specific to *Sox10* expression and was not observed with *Ret*. (B) RA treatment was associated with increased *Sox10* levels but EDN3 had no effect at 14 days. (C) RA decreased *Ret* levels at 3 days regardless of whether EDN3 was present, but EDN3 did not affect *Ret* gene expression. (D) Decreased *Ret* levels in response to RA persisted at 14 days, but only in the presence of EDN3

SOX10 expression is associated with ENS precursor maintenance and glial differentiation [38–41]. EDN3 maintains SOX10 levels *in vivo* and SOX10 regulates EDN signaling by acting directly on the *Ednrb* promoter [42,43]. We therefore wanted to assess the relationship of EDN and *Sox10* expression in the relatively controlled environment of our defined culture system and determine whether RA impacts this relationship (Figure 2 and Table 4). RA and EDN3 interacted in the regulation of *Sox10*: In the absence of EDN3, RA doubled *Sox10* levels at 3 days (p=0.003). However, the concurrent addition of EDN3 abolished this phenomenon. After 14 days, RA was still associated with a 2- to 6-fold increase in *Sox10* abundance, but this association was no longer affected by EDN3 *Sox10* levels sharply declined by 84% (p=0.029) to 94% (p<0.001) in response to EDN3, depending on whether RA was also present. These data reveal a bi-directional interaction between EDN3 and RA in the modulation of *Sox10*, with RA favoring, and EDN3 antagonizing *Sox10* expression.

**Table 4:** Changes in ENS development-related mRNA levels in response to RA and EDN3.

	Comparison of relative mean mRNA levels			
	(p value)			
Target gene:	*Ret*		*Sox10*	
	3d	14d	3d	14d
**RA- vs. RA+ (EDN3-)**	**1.0 vs. 0.356**	1.0 vs. 0.560	**1.0 vs. 2.267**	**1.0 vs. 2.116**
	**(p<0.001)**		**(p=0.003)**	**(p=0.012)**
**RA- vs. RA+ (EDN3+)**	**0.881 vs. 0.327**	**1.286 vs. 0.419**	0.160 vs. 0.127	**0.423 vs. 2.613**
	**(p<0.001)**	**(p=0.032)**		**(p<0.001)**
**EDN3- vs. EDN3+ (RA-)**	1.0 vs. 0.881	1.0 vs. 1.286	**1.0 vs. 0.160**	1.0 vs. 0.423
			**(p=0.029)**	
**EDN3- vs. EDN3+ (RA+)**	0.356 vs. 0.327	0.560 vs. 0.419	**2.267 vs. 0.127**	2.116 vs. 2.613
			**(p<0.001)**	
**Interaction present?**	No	No	**Yes (p=0.019)**	No

This table summarizes the effects of RA and EDN3 on the gene expression in ENS precursor cultures after 3 and 14 days. Immunoselected cells were cultured in the presence of RA, EDN3, RA with EDN3, or neither compound. When EDN3 was absent, the EDN signaling inhibitor BQ-788 was added to inhibit endogenous EDNRB signaling. Relative mRNA levels were measured by quantitative RT-PCR. Levels are normalized to β-actin and the - RA/- EDN3 condition was standardized to a value of 1. Relative mRNA levels were compared between the groups. A two way ANOVA was employed to identify the contribution of each compound to changes in the proportion of proliferating cells. Significant changes (p<0.05) are in bold. Abbreviations: 3d = 3-day culture; 14d = 14-day culture; EDN3 = endothelin-3; RA = Retinoic acid;

### RA exerts a stable suppressive effect on Ret expression

Since *Ret* deficiency is associated with ENS precursor defects and intestinal aganglionosis [44] we explored the possibility that EDN3 and/or RA also contribute to the transcriptional regulation of *Ret* in these cells. We cultured p75^NTR^+ cells for 3 and 14 days with different combinations of EDN3 and RA, as above, and mRNA levels were quantified. Although the addition of EDN3 did not significantly affect *Ret* gene expression, RA was associated with a 62% net decrease in *Ret* levels at 3 days (p<0.001) and 67% at 14 days (p=0.032) (Figure 2 and Table 4). With these data, RA may be added to the factors that regulate the transcription of *Ret* in ENS precursors, although further investigation is necessary to determine whether this occurs by direct action on the *Ret* gene or whether this reflects changes in the cell composition of the culture.

### RA is associated with a net decrease in EDN-related gene expression

**Figure 3:**
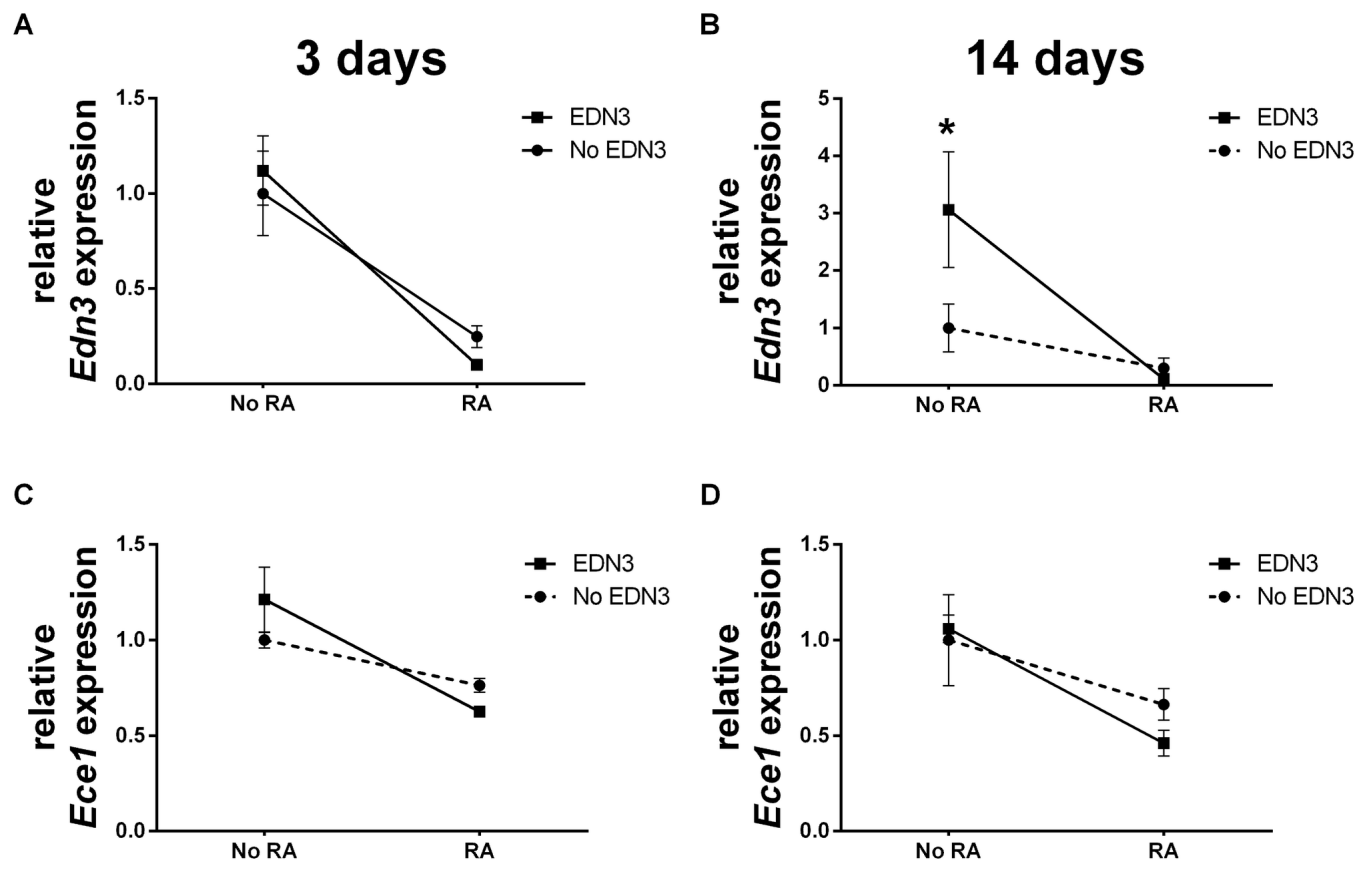
RA decreases *EDN3* and *Ece1* mRNA levels, depending on whether exogenous EDN3 is present. p75^NTR^+ cells were grown for 72 hours in the presence of RA, EDN3, RA with EDN3, or neither compound. When EDN3 was absent, the EDN signaling inhibitor BQ-788 was added to inhibit endogenous EDNRB signaling. Relative *EDN3* (A-B) and *Ece1* (C-D) mRNA levels were measured by quantitative RT-PCR after 3 (A, C) and 14 (B, D) days. Levels are normalized to β-actin and the -RA/-EDN3 condition was standardized to a value of 1. Solid lines denote a statistically significant difference (p<0.05) between “No RA” and “RA”, and broken lines indicate a p value >0.05. Asterisk denotes a statistically significant difference (p<0.05) between “No EDN3” and “EDN3”. (A) At 3 days, RA was associated with a net decrease in the level of *EDN3* in the absence and presence of exogenous EDN3 (C) Similarly, RA was associated with a decrease in the relative abundance of *Ece1*, albeit only in the presence of EDN3 After 14 days, RA still decreased the relative abundance of *EDN3* (B) and *Ece1* (D) mRNA.

In light of the opposing actions of EDN3 and RA on lineage predominance and *Sox10* expression in our *in vitro* system, we sought to determine how, if at all, RA and EDN3 affect each other at the gene expression level (Figure 3 and Table 5). We observed that the addition of RA was associated with a net decrease in the level of *EDN3* by 75% (p=0.004) in the absence of exogenous EDN3, and by 91% (p<0.001) in the presence of exogenous EDN3 (p<0.001). Similarly, adding RA was associated with a decrease in the relative abundance of *Ece1* by 48% (p<0.001), albeit only in the presence of EDN3 Neither RA nor EDN3 were associated with changes in the expression of the *Ednrb* gene at 3 days. Collectively, these data demonstrate that culture of ENS precursors in the presence of RA leads to a decrease in abundance of *EDN3* and *Ece1* mRNA.

**Table 5:** Changes in EDN-related mRNA levels in response to RA and EDN3.

	Comparison of relative mean mRNA levels					
	(p value)					
**Target gene:**	*EDN3*		*Ece1*		*Ednrb*	
	3d	14d	3d	14d	3d	14d
**RA- vs. RA+ (EDN3-)**	**1.0 vs. 0.248**	1.0 vs. 0.299	1.0 vs. 0.763	1.0 vs. 0.663	1.0 vs. 1.512	1.0 vs. 15.201
	**(p=0.004)**					
**RA- vs. RA+ (EDN3+)**	**1.12 vs. 0.099**	**3.062 vs. 0.110**	**1.213 vs. 0.625**	**1.059 vs. 0.461**	1.512 vs. 1.246	**2.8 vs. 22.873**
	**(p<0.001)**	**(p=0.006)**	**(p<0.001)**	**(p=0.014)**		**(p=0.017)**
**EDN3- vs. EDN3+ (RA-)**	1.0 vs. 1.120	**1.0 vs. 3.062**	1.0 vs. 1.213	1.0 vs. 1.059	1.0 vs. 1.512	1.0 vs. 2.8
		**(p=0.030)**				
**EDN3- vs. EDN3+ (RA+)**	0.248 vs. 0.099	0.299 vs. 0.110	0.763 vs. 0.625	0.663 vs. 0.461	1.553 vs. 1.246	15.201 vs. 22.873
**Interaction present?**	No	No	No	No	No	No

This table summarizes the effects of RA and EDN3 on the gene expression in ENS precursor cultures after 3 and 14 days. Immunoselected cells were cultured in the presence of RA, EDN3, RA with EDN3, or neither compound. When EDN3 was absent, the EDN signaling inhibitor BQ-788 was added to inhibit endogenous EDNRB signaling. Relative mRNA levels were measured by quantitative RT-PCR. Levels are normalized to β-actin and the - RA/- EDN3 condition was standardized to a value of 1. Relative mRNA levels were compared between the groups. A two way ANOVA was employed to identify the contribution of each compound to changes in the proportion of proliferating cells. Significant changes (p<0.05) are in bold. Abbreviations: 3d = 3-day culture; 14d = 14-day culture; EDN3 = endothelin-3; RA = Retinoic acid;

### EDN3 treatment leads to a net reduction in RA receptor gene expression

**Figure 4:**
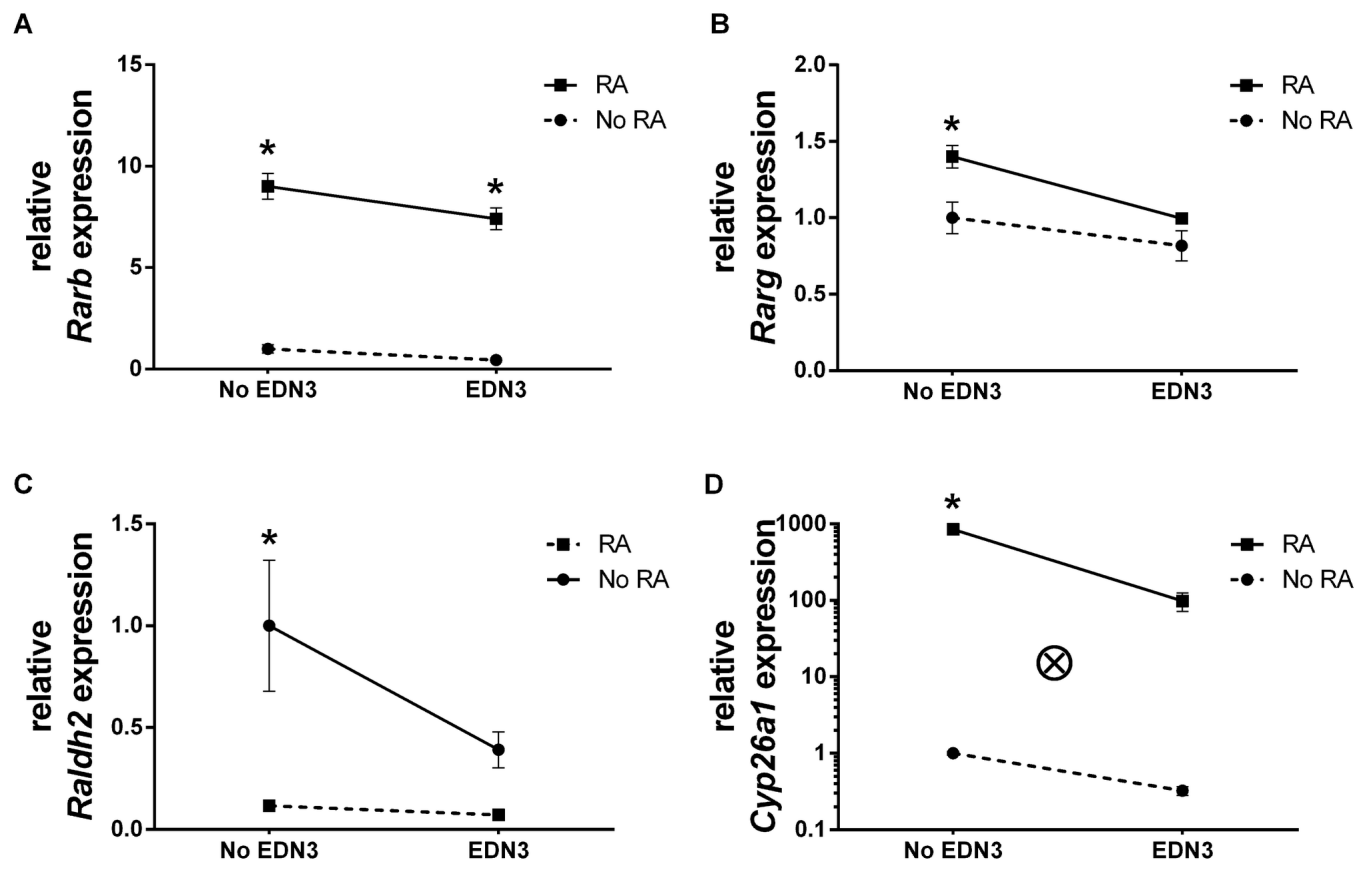
EDN3 is associated with decreased levels of RA receptor mRNA and RA metabolic enzyme mRNA. p75^NTR^+ cells were grown for 72 hours in the presence of RA, EDN3, RA with EDN3, or neither compound. When EDN3 was absent, the EDN signaling inhibitor BQ-788 was added to inhibit endogenous EDNRB signaling. Relative *Rarb* (A), *Rarg* (B), *Raldh2* (C), and *Cyp26a1* (D) mRNA levels were measured by quantitative RT-PCR. Levels are normalized to β-actin and the -RA/-EDN3 condition was standardized to a value of 1. Solid lines denote a statistically significant difference (p<0.05) between “No EDN3” and “EDN3”, and broken lines indicate a p value >0.05. Asterisk denotes a statistically significant difference (p<0.05) between “No RA” and “RA”. EDN3 was associated with a net decrease in *Rarb* (A) and *Rarg* (B) mRNA transcripts in the presence of exogenous RA. In the absence of exogenous RA, EDN3 did not have an effect on RA receptor levels. (C) EDN3 was also associated with a decrease in *Raldh2* abundance, but not when RA was present. (D) EDN3 also reduced *Cyp26a1* mRNA levels, but only in the presence of RA. Consistent with autoregulation, RA treatment was responsible for a reduction in *Raldh2* levels (C) and an increase in *Cyp26a1* (D), in the absence of exogenous EDN3 However, associations of RA with *Raldh2* and *Cyp26a1* levels were lost when EDN3 was simultaneously present. There was a statistically significant interaction (ⓧ; p<0.001) between EDN3 and RA in their effect on *Cyp26a1* levels at 3 days.

Since RA was associated with decreased abundance of *EDN3* and *Ece1* gene expression, we next examined whether the converse was true: Is EDN3 associated with changes in the expression of RA signaling related genes in 3-day cultures? EDN3 was associated with a net decrease in *Rarb* and *Rarg* RNA transcripts (Figure 4 and Table 6): in the presence of RA, EDN3 decreased *Rarb* and *Rarg* by 18% (p=0.023) and 28% (p=0.005), respectively. In the absence of RA, EDN3 did not contribute to RA receptor expression. Consistent with previous studies, adding RA was associated with increased levels of *Rarb* (p<0.001) and *Rarg* (p=0.005) RNA transcripts [45–54]. No significant effects on *Rara* were observed with either compound. These experiments suggest that EDN3 and RA competitively alter the abundance of *Rarb* and *Rarg*.

**Table 6:** Changes in RA receptor-related mRNA levels in response to RA and EDN3.

	Comparison of relative mean mRNA levels					
	(p value)					
**Target gene:**	*Rara*		*Rarb*		*Rarg*	
	3d	14d	3d	14d	3d	14d
**RA- vs. RA+ (EDN3-)**	1.0 vs. 0.953	1.0 vs. 1.124	**1.0 vs. 9.005**	**1.0 vs. 183.758**	**1.0 vs. 1.4**	1.0 vs. 0.854
			**(p<0.001)**	**(p=0.002)**	**(p=0.005)**	
**RA- vs. RA+ (EDN3+)**	0.846 vs. 1.028	1.315 vs. 0.751	**0.438 vs. 7.406**	**1.589 vs. 222.325**	0.817 vs. 0.995	1.320 vs. 1.031
			**(p=0.001)**	**(p<0.001)**		
**EDN3- vs. EDN3+ (RA-)**	1.0 vs. 0.846	1.0 vs. 1.315	1.0 vs. 0.438	1.0 vs. 1.589	1.0 vs. 0.817	1.0 vs. 1.32
**EDN3- vs. EDN3+ (RA+)**	0.953 vs. 1.028	1.124 vs. 0.751	**9.005 vs. 7.406**	183.758 vs. 222.325	**1.4 vs. 0.995**	0.854 vs. 1.031
			**(p=0.023)**		**(p=0.005)**	
**Interaction present?**	No	No	No	No	No	No

This table summarizes the effects of RA and EDN3 on the gene expression in ENS precursor cultures after 3 and 14 days. Immunoselected cells were cultured in the presence of RA, EDN3, RA with EDN3, or neither compound. When EDN3 was absent, the EDN signaling inhibitor BQ-788 was added to inhibit endogenous EDNRB signaling. Relative mRNA levels were measured by quantitative RT-PCR. Levels are normalized to β-actin and the - RA/- EDN3 condition was standardized to a value of 1. Relative mRNA levels were compared between the groups. A two way ANOVA was employed to identify the contribution of each compound to changes in the proportion of proliferating cells. Significant changes (p<0.05) are in bold. Abbreviations: 3d = 3-day culture; 14d = 14-day culture; EDN3 = endothelin-3; RA = Retinoic acid;

### EDN3 and RA regulate RA metabolic enzyme gene expression in a complex fashion

RA homeostasis is regulated, in part, by the RA synthesizing enzyme RALDH2, and the RA degrading enzyme CYP26A1 [18]. Since EDN3 was shown to reduce the abundance of RA receptor mRNA, we next explored the possibility that EDN3 alters the availability of RA by affecting *Raldh2* and *Cyp26a1* expression *in vitro* (Figure 4 and Table 7). The responses of these genes to EDN3 and RA were complex. EDN3 was associated with a 61% (p=0.024) decrease in *Raldh2* abundance, but this decrease was abolished when RA was present. EDN3 also significantly reduced *Cyp26a1* mRNA levels by 88% when RA was added concomitantly (p<0.001). RA treatment was responsible for a reduction by 88% in *Raldh2* levels (p<0.003) and a >800-fold increase in *Cyp26a1* (p<0.001), in the absence of exogenous EDN3 However, a statistically significant association of RA with *Raldh2* and *Cyp26a1* levels were not observed when EDN3 was simultaneously present, suggesting that EDN3 somehow disrupts this process. Together, these findings suggest that EDN3 is capable of impacting RA levels via an effect on the gene expression of RA metabolic machinery.

**Table 7:** Changes in RA metabolism enzyme-related mRNA levels in response to RA and EDN3.

	Comparison of relative mean mRNA levels			
	(p value)			
Target gene:	*Raldh2*		*Cyp26a1*	
	3d	14d	3d	14d
**RA- vs. RA+ (EDN3-)**	**1.0 vs. 0.117**	**1.0 vs. 4.748**	**1.0 vs. 846.015**	1.0 vs. 1.682
	**(p=0.003)**	**(p=0.007)**	**(p<0.001)**	
**RA- vs. RA+ (EDN3+)**	0.391 vs. 0.072	0.619 vs. 0.257	0.325 vs. 98.403	**0.139 vs. 6.461**
				**(p<0.001)**
**EDN3- vs. EDN3+ (RA-**)	**1.0 vs. 0.391**	1.0 vs. 0.619	1.0 vs. 0.325	1.0 vs. 0.139
	**(p=0.024)**			
**EDN3- vs. EDN3+ (RA+)**	0.117 vs. 0.072	**4.748 vs. 0.257**	**846.015 vs. 98.403**	**1.682 vs. 6.461**
		**(p=0.003)**	**(p<0.001)**	**(p=0.004)**
**Interaction present?**	No	**Yes (p=0.023)**	**Yes (p<0.001)**	**Yes (p=0.01)**

This table summarizes the effects of RA and EDN3 on the gene expression in ENS precursor cultures after 3 and 14 days. Immunoselected cells were cultured in the presence of RA, EDN3, RA with EDN3, or neither compound. When EDN3 was absent, the EDN signaling inhibitor BQ-788 was added to inhibit endogenous EDNRB signaling. Relative mRNA levels were measured by quantitative RT-PCR. Levels are normalized to β-actin and the - RA/- EDN3 condition was standardized to a value of 1. Relative mRNA levels were compared between the groups. A two way ANOVA was employed to identify the contribution of each compound to changes in the proportion of proliferating cells. Significant changes (p<0.05) are in bold. Abbreviations: 3d = 3-day culture; 14d = 14-day culture; EDN3 = endothelin-3; RA = Retinoic acid;

### The relationship of EDN and RA signaling and metabolism changes as ENS precursors differentiate

Our next objective was to determine the changes in gene expression in later cultures and correlate these with earlier expression changes and phenotypic changes. We evaluated the expression of EDN- and RA-related genes after 14 days (Tables 4-7). *EDN3* and *Ece1* abundance continued to be lower in cultures treated with RA, with decreases of 96% (p=0.006) and 56% (p=0.014), respectively, when exogenous EDN3 was concurrently present (Figure 3). In contrast to observations in our short term cultures where RA was not associated with *Ednrb* expression, we observed an 8-fold increase (p=0.017) in *Ednrb* transcript levels in response to RA treatment (Table 5). The relationship between EDN3 and *Rar* expression that was seen at 3d was not observed at 14 days (Figure 4). Nevertheless, RA, EDN3, and the combination of RA and EDN3 continued to have distinct significant effects on *Raldh2* and *Cyp26a1* levels indicating an interaction between the compounds in the modulation of gene expression: EDN3 was associated with a net 95% reduction (p=0.003) in *Raldh2* levels and net 3.8-fold increase in *Cyp26a1* when RA was also present. RA augmented *Raldh2* levels almost 5-fold at 14 days, but this relationship was abolished when EDN3 was concomitantly present. RA was associated with a 45-fold increase in *Cyp26a1* expression (p<0.001), but only in the presence of EDN3 Collectively, these data reflect the existence of a bi-directional interaction between EDN3 and RA in the modulation of RA-related gene expression. We hypothesize that this interaction occurs *in vivo* and impacts the local levels of bioavailable RA*.*

### RA induces the formation of an organized heterocellular plexus

**Figure 5:**
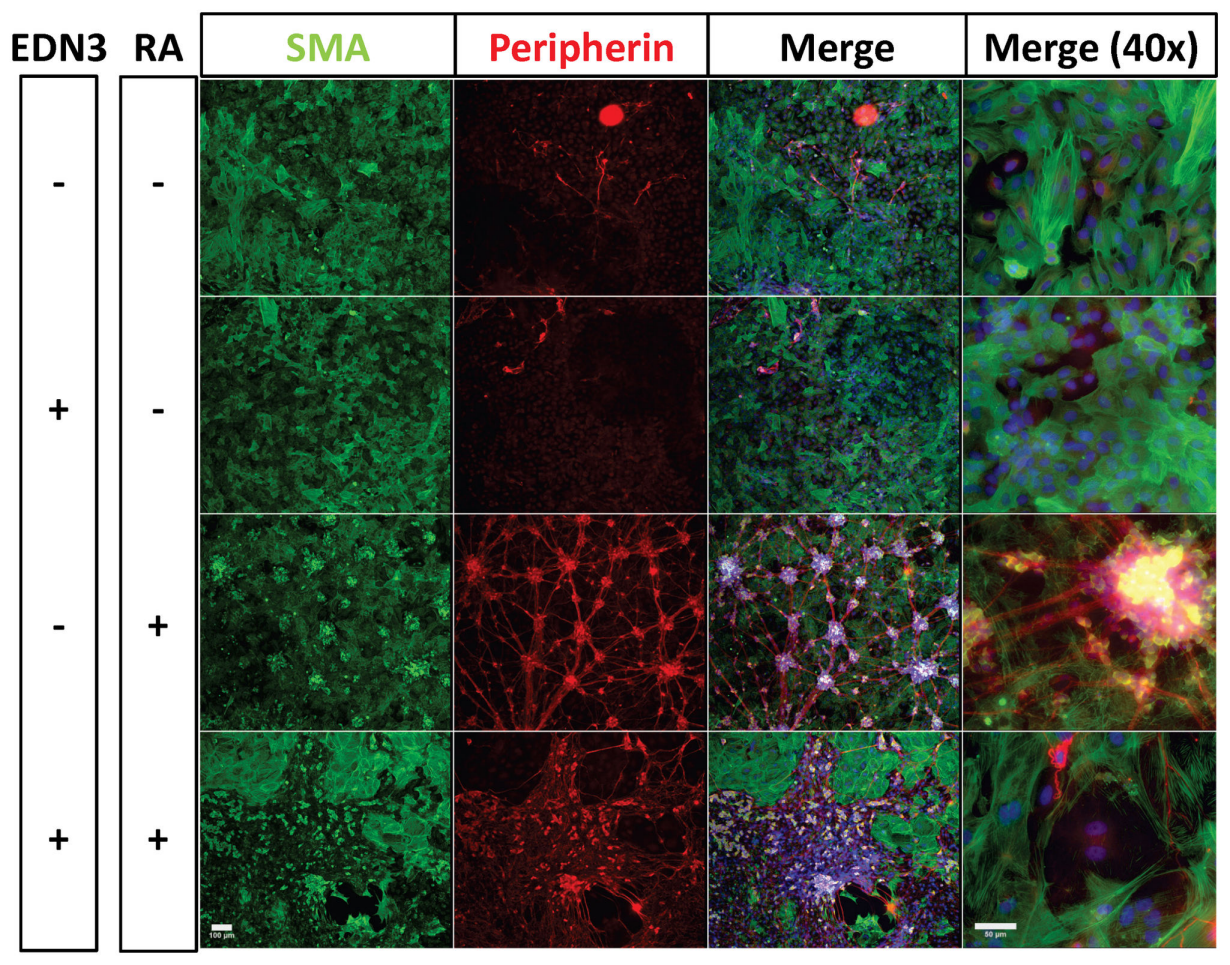
Effects of RA and EDN3 on the terminal culture morphology of ENS precursors. p75^NTR^ immunoselected cells were grown for 14 days in the presence of RA, EDN3, RA with EDN3, or neither compound. When EDN3 was absent, the EDN signaling inhibitor BQ-788 was added to inhibit endogenous EDNRB signaling. Culture morphology was evaluated by immunofluorescence microscopy. Peripherin expression (red) is consistent with neuronal morphology and myofibroblasts are indicated by expression of α-SMA (green). The third and fourth columns include DAPI staining for nuclei. The first three columns were photographed at 10x (Scale bar = 100 µm) and the fourth column was photographed at 40x (Scale bar = 50 µm). In the absence of EDN3 and RA (EDN3-RA-), SMA+ myofibroblasts predominated, but sparse neurons were also seen. EDN3+RA- treated cultures formed a homogeneous sheet of SMA+ myofibroblasts. In EDN3-RA+ cultures, neurons formed a plexus punctuated by large multicellular ganglia, abundant peripherin+ neurites and cell bodies, and non-myofibroblast-like SMA+ cells. SMA+ myofibroblasts were also present beneath the plexus. With EDN3+RA+ treatment, many peripherin+ neurons and long complex neurites were seen, without forming a plexus. Myofibroblasts, while excluded from neuronal regions, were still present in large number.

**Figure 6:**
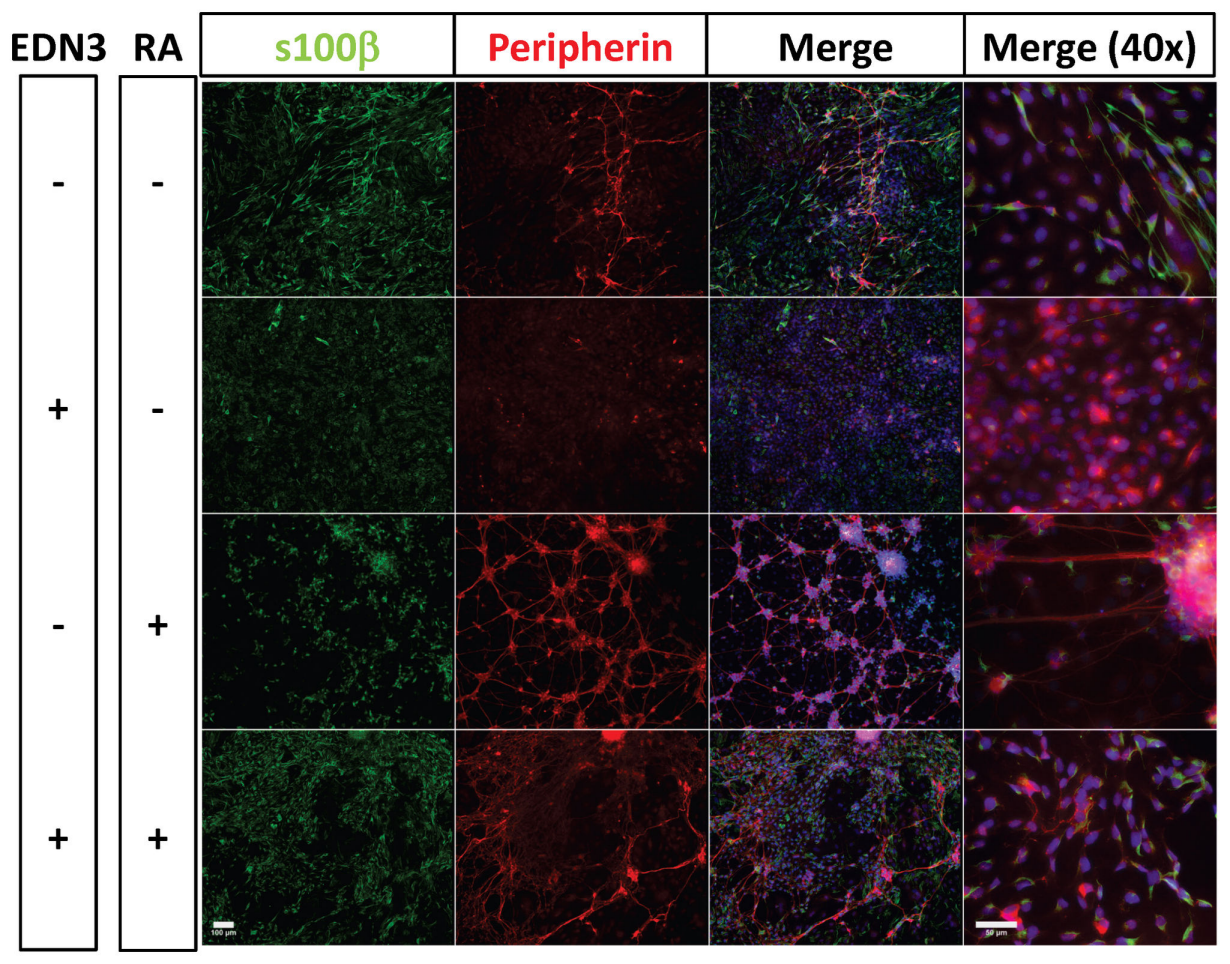
Effects of RA and EDN3 on the terminal culture morphology of ENS precursors. p75^NTR^ immunoselected cells were grown for 14 days in the presence of RA, EDN3, RA with EDN3, or neither compound. When EDN3 was absent, the EDN signaling inhibitor BQ-788 was added to inhibit endogenous EDNRB signaling. Culture morphology was evaluated by immunofluorescence microscopy. Peripherin expression (red) is consistent with neuronal morphology and glia are indicated by expression of S100β (green). The third and fourth columns include DAPI staining for nuclei. The first three columns were photographed at 10x (Scale bar = 100 µm) and the fourth column was photographed at 40x (Scale bar = 50 µm). Without EDN3 and RA (EDN3-RA-) sparse neurons developed, but S100β+ glia were more numerous and more brightly staining. EDN3+RA- cultures did not contain glia or neurons. EDN3-RA+ cultures formed discrete heterocellular ganglia with abundant peripherin+ cell bodies and S100β+ glia, linked by thick peripherin+ neurites in a plexus pattern. EDN3+RA+ treatment also contained many peripherin+ neurons and long complex neurites, but these neurons were disorganized, and did not form a plexus. Weakly staining S100β+ cells with glial cell morphology were also found amidst the neurons. The glia in these cultures were more fusiform and were mainly associated with neurons.

To understand how the changes in S100β+ cell prevalence induced by RA and EDN3 relate to terminal culture morphology, we studied ENS precursor cultures after 14 days (Figures 5 and 6). High cell densities, as well as the 3-dimensional structure of these cultures precluded accurate quantitative comparisons of the different lineages. Nevertheless, distinct features of each condition were evident. Cells cultured in the absence of EDN3 and RA (EDN3-RA-) were predominantly myofibroblastic in nature, although they did develop sparse neurons, and more numerous and more brightly staining glia. EDN3+RA- cultures formed an almost-uniform amorphous sheet of SMA+ cells possessing myofibroblast morphology. EDN3-RA+ cultures were distinct in that neurons formed a plexus pattern, punctuated by large heterocellular ganglia, abundant peripherin+ neurites and cell bodies, abundant S100β+ cells, and non-myofibroblast-like SMA+ cells. Cells morphologically resembling myofibroblasts were also abundantly present, but resembled a feeder monolayer beneath the plexus. EDN3+RA+ treatment resulted in the emergence of many peripherin+ cells with neuronal morphology and long complex neurites, but these neurons were disorganized, and did not form a discernible plexus. Furthermore, myofibroblasts, while excluded from neuronal regions, were still present in large number. Other SMA+ cells were also observed among the neurons, but lacked myofibroblast morphology and the lineage of these cells is unclear. Weakly staining S100β+ cells with glial cell morphology were also found amidst the neurons. The glia in these cultures were more fusiform and were mainly associated with neurons. Overall, RA presence supported the formation of neurons and glia but activation of EDNRB in these same cultures resulted in a significant difference in the arrangement of the neuronal elements.

## Discussion

Both RA and EDNRB signaling play critical roles in the formation of the ENS. Evidence from disparate sources [5,20] suggests that the two morphogens spatiotemporally overlap during ENS development. Furthermore, RA has been shown to modulate EDN signaling in other cell types [30–33], although the reverse has not been demonstrated. The fact that both are implicated in the pathogenesis of aganglionosis prompted us to investigate their combined effects and to determine whether there exists a relationship between the two pathways in ENS precursor cultures. Our findings suggest that 1) RA supports enteric glial proliferation resulting in an increased abundance of enteric glia and decreased neurons in culture, 2) EDNRB signaling depletes enteric glia by suppressing their proliferation but promotes proliferation overall, 3) RA suppresses *Ret* gene expression and this is associated with a decreased proportion of neurons in culture, 4) RA signaling is associated with a decrease in the abundance of *EDN3* mRNA, 5) EDN3 alters the abundance of mRNA encoding RA receptors and metabolic enzymes, and 6) RA and EDNRB signaling interact to affect the morphology of mature cultures of ENS precursors *in vitro*.

Enteric neurons develop from neural crest-derived precursors that persist in the adult gut and share many properties with enteric glia, including the expression of SOX10, and p75^NTR^ [55,56]. Multipotent ENS precursors and mature glia also express EDNRB [13,57–61]. The distinction between neurogenic precursors and enteric glia has been blurred by the striking finding that enteric glia are capable of undergoing neurogenesis [55,56,62]. Our data demonstrate that RA stimulates proliferation of S100β+ cells at 3 days, resulting in increased abundance of these cells. Whether these cells represent mature glia or a specialized stem cell population is unclear. Interestingly, *Sox10*, but not *Ednrb*, expression is also increased after 3 days in the presence of RA. Increased *Sox10* expression is consistent with a study showing a dose-dependent positive effect of RA on chimeric SOX10-GFP protein expression in murine embryonic stem cells, resulting in the acquisition of markers of neural crest progenitors [63]. If we interpret the lack of a concomitant increase in the EDNRB+ population to indicate stable numbers of ENS progenitors, our results suggest that the increase in S100β and *Sox10* expression observed in the presence of RA reflects an increase in the number of mature glia. Culture in the presence of the EDNRB ligand dramatically suppresses *Sox10* expression, even in the presence of RA, revealing a significant statistical interaction in the relationship of RA and EDN3 with *Sox10* expression. The underlying biological mechanism for this association is unknown and will require further study. Nevertheless, effects on *Sox10* do not appear to be solely a reflection of a decreased percentage of S100β+ glia in the culture because *Sox10* expression is reduced even in EDN3+RA+ cultures, where S100β+ cells are increased. Previous studies demonstrate that EDNRB activation of ENS progenitors induces proliferation of a SOX10+ cell population [5]. One explanation for our findings is that over the course of the three day culture, in which secondary and tertiary effects may occur, EDN3-RA+ culture promotes the development of SOX10+S100β+ cells while EDN3+RA+ culture promotes the development of SOX10-S100β+ cells. These two populations may represent distinct subpopulations of enteric glia. Joseph et al. observed that 26% of S100β+ gut cells expressed the ENS stem cell marker integrin alpha2 and were capable of forming multilineage neurospheres in culture [55]. They hypothesized that enteric glia may be heterogeneous in their ability to form neurons. While it is established the multipotent ENS precursors express EDNRB, EDNRB expression in subpopulations of cells along the neural and glial differentiation pathways has not been studied making conclusions regarding the lack of change of EDNRB expression in response to RA difficult to interpret. RA does not affect the total number of S100β+RET+ mouse p75^NTR^-immunoselected ENS precursors after 7 days in culture [21]. It is likely that the S100β+ cells we studied are a different population. RA treatment was also associated with a statistically significant decrease in the abundance of peripherin+ cells bearing neuronal morphology. This is surprising because RA increases neuronal differentiation in other neuronal lineages [21,64–67]. As such, we speculate that alterations in the proportion of neurons are a reflection of relatively increased glial abundance rather than a direct effect of RA on neurogenesis. Since the proportion of each lineage was measured separately, we could not discern the relative ratios of each lineage, so appraisal of the effects of RA on neurogenesis in this system requires further study.

Contrasting our observations with those of Sato and Heuckeroth [21], our RA-induced decrease in neuronal abundance may reflect subtle but important differences in our culture method. Specifically, there were differences in culture medium components (neurobasal vs. DMEM), RA concentration (10^-6^ vs. 10^-7^M), and oxygen concentration during culture incubation (21% vs. ~5%). As the half-life of RA and its metabolites is affected by these parameters, it is reasonable to assume that conditions that most closely match physiologic conditions *in utero* better reflect the underlying biology. The limitations of reductionist *in vitro* systems highlight the necessity for further *in vivo* work to shed light on how retinoid deficiency or excess might affect the developing ENS.

In contrast to RA, we found that EDN3 decreases S100β+ cell proliferation and decreases their abundance in 3-day cultures. Our data contrast with another study showing that EDN3 promotes the expansion of glia from multipotent precursors. However, the cells assayed were derived from quail trunk neural crest at an earlier developmental stage, and a distinct glial marker (Schwann Cell Myelin Protein) was employed [68]. Furthermore, enteric glia express functional EDNRB receptors and elaborate EDNs, and rats in which functional EDNRB receptors are absent feature accelerated expression of S100 (a Schwann cell marker) in developing nerves [61,69]. This aligns with our observation that EDN3 acts as negative regulator of S100β-expressing cells. Despite effects on glial abundance, EDN3 did not affect proliferation or abundance of neurons or myofibroblasts in culture.

In addition to lineage-specific proliferative changes, we also observed an increase in the overall proportion of proliferating cells in response to RA and in response to EDN3 While the increase in response to RA treatment may be attributable to proliferation amongst the glial lineage discussed above, we did not identify any lineage-specific increases in response to EDN3 in our experiments. As such, we may infer that the global increase in proliferation in response to EDN3 also occurs subtly in one or more specific lineages, but below the threshold of statistical significance, or occurs in a population not detected by us, i.e. peripherin-SMA-S100β- cells. The nature of this population is unclear, but based on previous work we can speculate that these cells may be immature neural crest-derived cells, as EDN3 has been shown to stimulate proliferation of enteric neuronal precursors under similar culture conditions [5,10,11].

The RET receptor is expressed on proliferating neurogenic and gliogenic precursors and several proteins, including SOX10, are known to regulate its transcription [70,71]. These precursors continue to express RET as they differentiate into neurons but lose RET expression if they differentiate into glia [35,72–74]. EDN and RET signaling act synergistically to permit normal development of the ENS [17,75] and interaction probably occurs at the level of common downstream intracellular signals [5]. The effect of RA on *Ret* expression is less clear and likely depends on the model system being studied. RA is known to be a potent activator of *Ret* in neuroblastoma cell lines [76–78], but down-regulates *Ret* in models of cardiac development [79]. Niederreither et al. observed that deletion of *Raldh2* is associated with a decrease in *Ret*-expressing neuroblasts, suggesting that RA is necessary to maintain *Ret* expression or *Ret*-expressing cells [29]. In a murine cell population resembling ours, RA was shown to stimulate the proliferation of a subpopulation of RET+ neuroblasts [21]. We observed a decrease in *Ret* mRNA levels in RA-treated ENS precursor cultures. These data are consistent with the gliogenic effect and the decline in peripherin+ cells in our 3-day cultures in response to RA. Curiously, an RA-induced decrease in *Ret* mRNA was also noted in 14-day cultures coinciding with an RA-induced increase in neuronal abundance. This contrasts with an RA-associated increase in the abundance of RET+TUJ+ neurons in a similar model [21]. Our data therefore suggest that our culture conditions (which differ from those used by Sato and Heuckeroth – see above) are more conducive to the development of RET-negative neurons. Alternatively, *Ret* gene expression is likely not proportional to RET surface protein expression.

As glia and *Sox10* appear to be responsive to both RA and EDN3 signaling, we hypothesized that the two signals could reciprocally control each other. Since RA modulates EDN signaling in other contexts, we sought this phenomenon in ENS precursor cultures [30–33]. RA treatment decreased *EDN3* and the EDN3 activating enzyme, *Ece1* mRNA levels in our ENS precursor cultures. The mechanism by which RA modulates *EDN3* and *Ece1* expression is unclear. Direct transcriptional repression is unlikely because the *EDN3* and *Ece1* promoters are not purported to contain canonical RA response elements (RAREs). Nevertheless, it is possible that RA regulates expression via non-canonical RAREs, directly via RA receptor independent mechanisms, or indirectly via other intermediates. However, given the increase in s100β+ cells in response to RA, a more plausible explanation is that the glia we studied scarcely express *EDN3*/*Ece1* and their expansion is therefore associated with a net decrease in *EDN3*/*Ece1*. While *EDN3* transcript levels remained low in the differentiated 14 day cultures, *Ednrb* transcripts are significantly increased by culture in the presence of RA. The increase in *Ednrb* transcript levels in 14 day cultures may be the result of long-term RA suppression of EDN3 resulting in a compensatory increase in *Ednrb* expression or may be related to altered phenotypic differentiation. Alternatively, the increased abundance of *Ednrb* reflects the expansion of enteric glia and neurons in RA-treated cultures, both of which are known to express this receptor [43,61]. Further study of the expression of ECE1, EDRNB, EDN3 and RA-related proteins will be necessary to address these issues.

Our defined culture system induced ectopic *EDN3* in ENS precursors after 3 days in culture, which differs from the *in vivo* observation that EDN3 is expressed by the developing gut mesenchyme, not by ENS precursors [5,9,10]. Although our cultures are enriched for immunoselected p75^NTR^+ ENS precursors, and the media preferentially supports the growth of these cells, the cultures contain a minority of p75^NTR^- cells which may be gut mesenchymal cells that express EDN3 However, it is unlikely that the addition of RA to the culture decreases *EDN3* expression solely by reducing the percentage of mesenchymal cells in the culture. If this were the case, we would expect an increase in many or all of the other markers of ENS precursors or their derivatives. Instead, RA treatment results in a reduction in *Ret* expression and no change in *Ednrb* or *Sox10* expression (except in the absence of EDNRB activation) in 3 day cultures. Further, we did not find an increase in the percentage of cells not labeling with peripherin or S100β (Table 2). Though we think it is unlikely, we cannot eliminate the possibility that RA down-regulates *EDN3* expression in the mesenchymal p75^NTR^- cell population. If true, this explanation for the expression of *EDN3* in our cultures and its down-regulation by RA supports an interaction of RA and EDNRB signaling in the expression of Hirschsprung’s disease, albeit in a non-neural crest autonomous fashion. Alternatively, vagal crest-derived cells are capable of changing their phenotype in culture, and the expression of *Edn3 in vitro* may reflect the influence of the culture environment [80]. For example, our data suggest that if *in vivo* microenvironmental levels of RA are high, *EDN3* expression by ENS precursors may be undetectable.

Until now, an influence of EDN3 on RA signaling has not been reported. We now demonstrate that EDN3 treatment reduces *Rarb* and *Rarg* levels in ENS precursor cultures. There are two ways that this may be achieved. First, EDN3 may reduce the abundance of ENS precursors that are known to express these receptors [21]. Alternatively, EDN3 treatment may affect transcript levels of these genes. The effects of EDN3 on *Raldh2* and *Cyp26a1* were more difficult to interpret: In the presence of RA, EDN3 decreased *Cyp26a1* levels at 3 days and increased levels at 14 days. EDN3 decreased *Raldh2* at 3 days and 14 days, but these phenomena were in association with the absence and presence of RA, respectively. We surmise that the diverse effects of EDN3 on the expression of RA metabolic machinery at different time points and conditions reflect a broader regulatory role for EDN3 in fine-tuning local levels of RA by interfering with RA feedback autoregulation. For instance, the EDN3 induced decrease in *Cyp26a1* expression may be a response to the decrease in RA signaling via the RA receptors. Indeed, previous work by others has suggested a link between RA receptor activation and *Cyp26a1* expression [81,82]. Moreover, after 14 days of culture, EDN3 may act as a brake on RA production by suppressing *Raldh2*. An alternative interpretation is that the emergence of diverse cell populations in extended cell culture is itself a cause for altered mRNA levels. Nevertheless, some observed gene expression changes occurring early in the culture period persisted after 14 days, despite the dramatic morphologic changes that occurred. Lastly, it should be recalled that the metabolism of RA is complex and many of its synthetic intermediates and degradation products are themselves bioactive [19,83,84]. The culture medium we used was lacking Vitamin A, an important RA precursor, thus many RA metabolites were likely also lacking. Although we have taken a reductionist approach to study the effects of RA and EDN3, it is our expectation that these studies will be complemented with *in vivo* and *in situ* models.

We observed four phenotypes in long-term cultures of ENS precursors that were dependent on the balance of EDN3 and RA: In the absence of RA, EDNRB activation led to exclusive myofibroblast differentiation and EDNRB signaling blockade allowed the emergence of glia in addition to myofibroblasts. RA supports the development of myofibroblasts, neurons and glia but with a strikingly different terminal morphology depending upon the presence or absence of EDNRB signaling. RA in the presence of EDNRB blockade supported the development heterocellular ganglia and a dense interconnected plexus. These findings may have implications regarding the molecular mechanisms that dictate the formation of the ENS in health and disease. It should be noted that long-term changes in cell phenotype, culture morphology, and gene expression were presumed to be attributable to the presence of RA and/or EDN3 However, since the study was limited to a single treatment of RA and/or EDN3 at the beginning of the culture period and the stability of EDN3 and RA in these long-term cultures is unknown, it is also possible that the observed changes are related to changes in the bioavailability of EDN3 and RA over time or that EDN3 and RA initiate specific transcriptional programs that persist in culture regardless of drug stability.

Consistent with previous reports [15,85], we found that EDN3 promoted the differentiation of ENS precursors into myofibroblasts as evidenced by their morphology and by smooth muscle actin immunoreactivity. Interestingly, the addition of RA supported the formation of neuronal and glial cells, in addition to myofibroblasts. Our data suggest that RA and EDN3 compete to promote specific lineages, with RA favoring neuronal/glial and EDN3 favoring myofibroblast formation in culture, while favoring the maintenance of an undifferentiated cell *in vivo*. This dichotomous differentiation pattern seen *in vitro* can arise by two distinct mechanisms: The first possibility is that RA and EDN3 each independently induce different lineages and the pattern that arises is dose-dependent. Alternatively, reciprocal inhibition of the opposing pathway may occur, signifying an interaction between the two signaling pathways. In order to discriminate between these two possibilities, we evaluated the morphological characteristics of cultures lacking EDN3 and RA, by simultaneously blocking EDNRB signaling and by not adding RA. Here, although the pattern was primarily myofibroblastic in nature, scant neurons were present and mature glial were represented more prominently. These data suggest that the default pathway of the ENS precursors in this culture system is permissive for myofibroblasts and glia, that EDN3 inhibits glial and neuronal differentiation, and that RA actively promotes neuronal (at 14 days) and glial differentiation.

**Figure 7:**
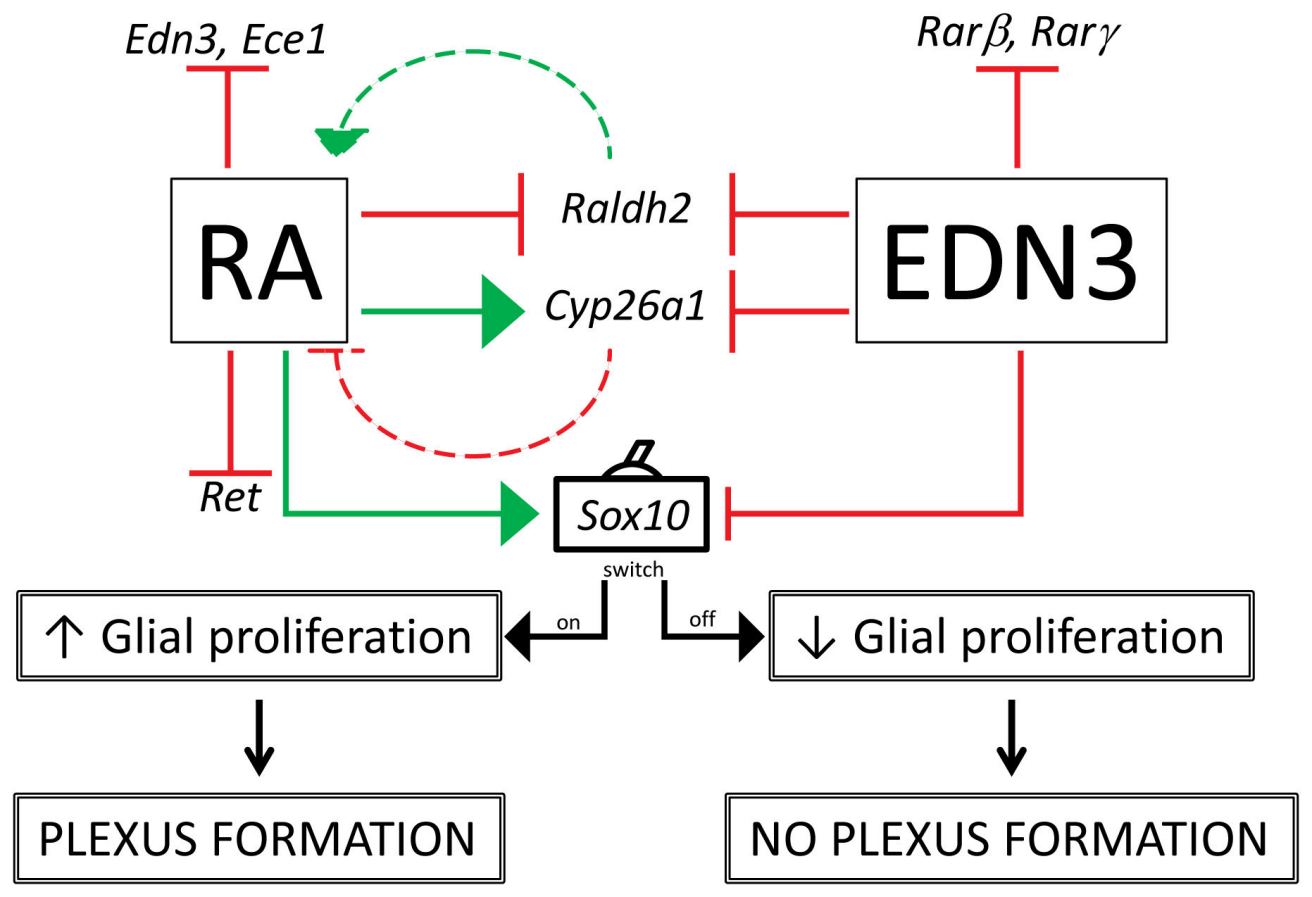
A hypothetical model depicting the bidirectional relationship of RA and EDN3 signaling in ENS precursors. The above pictogram, based on our data, summarizes and integrates the observed RNA expression profiles and the culture morphological data. Solid green arrows indicate increases in mRNA levels and solid red arrows with flat arrowheads denote decreases in mRNA levels. Although RA and EDN3 levels were not quantified in our culture model, dashed green and red arrows denote putative RA synthesis and degradation, respectively. We hypothesize that RA inhibits EDN signaling in ENS precursors by modulating the gene expression of *EDN3* and *Ece1*, and EDN3 regulates RA signaling by inhibiting RA receptor expression, thereby decreasing RA responsiveness, and fine-tuning RA metabolic enzyme expression, putatively altering RA availability. We propose that RA perpetuates glia by decreasing *Ret* and enhancing *Sox10* expression, and that EDN3 prevents the proliferation of glia by decreasing *Sox10*. The persistence of glia is conducive to the formation of a heterocellular ganglionated plexus. This model suggests that control of *Sox10* marks a key developmental decision point - a switch - that sustains multipotent progenitors in culture and ultimately depends on the balance of RA and EDN3

Taken together, our long-term culture data support the existence of a reciprocal relationship between RA and EDN3 (Figure 7). This relationship may underlie some of the opposing effects that RA and EDN3 exert on ENS precursors. For instance, activation of EDNRB signaling during the development of the ENS has been associated with the inhibition of neuronal differentiation, a decrease in self-renewal and impeded migration of ENS precursors *in vitro* [11,15]. Conversely, RA favors neurogenesis, migration and ENS precursor proliferation [21,28]. The potential implications of a bidirectional interaction are noteworthy: If RA is capable of lowering the overall pool of EDN3 in the developing gut, then changes in local RA concentrations may be responsible for the tightly-regulated spatiotemporal expression pattern of EDN3 It is also tempting to speculate that since EDN3 modulates *Cyp26a1* and *Raldh2* expression *in vitro*, and the proteins encoded by these genes are important in establishing RA gradients *in vivo*, EDN3 may play a role in the *in vivo* maintenance of an RA gradient in the gut [26,86,87]. Furthermore, based on our data, it is possible that RA, by effectively eliminating cell stimulation by EDN3, induces intrinsic changes in cells that permit them to differentiate, migrate, and proliferate.

The formation of ganglia is deemed to be important for the establishment of functional circuits. RA, in concert with EDN signaling blockade, induced the formation of a distinct plexus. Notably, while the addition of EDN3 did not inhibit differentiation of the precursors, it did inhibit the formation of this plexus, suggesting that EDN3 inhibits the RA-induced clustering of neuron cell bodies and glia into ganglia. The formation of a plexus requires differentiation of precursors into glial and neuronal components, neurite and axonal elongation, 3-dimensional spatial arrangements of its constituents, the establishment of synaptic connectivity and neuronal subtype specification [88–92]. Very little is known about the molecular mechanisms controlling each of these processes. Furthermore, there are few models for studying enteric plexuses *in vitro*. We have developed another model of plexus formation that, by virtue of it being an *in vitro* culture from undifferentiated cells, is amenable to the study of many aspects of plexus development, structure and function. ENS precursors that are permitted to develop undisturbed in an hypoxic environment in the presence of RA and the EDNRB selective antagonist BQ-788 spontaneously developed into a plexus containing neurons, glia, and supportive tissue, whose 3-dimensional arrangement is very reminiscent of an authentic gastrointestinal neural plexus.

## Conclusion

A complex EDN-RA interaction exists that coordinately regulates the development of rat ENS precursors *in vitro*. These results suggest that environmental RA may modulate the expression of aganglionosis in individuals with endothelin mutations.
